# Personalised colorectal cancer screening strategies: Information needs of the target population

**DOI:** 10.1016/j.pmedr.2023.102325

**Published:** 2023-07-16

**Authors:** Esther Toes-Zoutendijk, Lucie de Jonge, Emilie C.H. Breekveldt, Ida J. Korfage, Juliet A. Usher-Smith, Iris Lansdorp-Vogelaar, Rebecca A. Dennison

**Affiliations:** a Department of Public Health, Erasmus MC University Medical Centre, Rotterdam, The Netherlands; b Department of Gastroenterology and Hepatology, Netherlands Cancer Institute-Antoni van Leeuwenhoek Hospital, Amsterdam, The Netherlands; c Primary Care Unit, Department of Public Health and Primary Care, University of Cambridge, Cambridge, UK

**Keywords:** Personalised cancer screening, Colorectal cancer, Information need, Participation, Cancer risk, Information provision

## Abstract

Prior faecal Hemoglobin (f-Hb) concentrations of a negative fecal immunochemical test (FIT) can be used for risk stratification in colorectal cancer (CRC) screening. Individuals with higher f-Hb concentrations may benefit from a shorter screening interval (1 year), whereas individuals with undetectable f-Hb concentrations could benefit from a longer screening interval (3 year). Individuals’ views on personalised CRC screening and information needed to make a well-informed decision is unknown. We conducted three semi-structured focus groups among individuals eligible for CRC screening (i.e. men and women aged 55 to 75) in the Netherlands. Thematic analysis was used to analyse participants’ information need on personalised CRC screening strategies. Fourteen individuals took part. The majority were positive about CRC screening and indicated that they would participate in personalised CRC screening. The rationale for a longer interval among those at lowest risk was, however, unclear for many. The preferred information on individual risk was variable: ranging from full information to only information on the personalised strategy without mentioning the risk. It was not possible to address everyone’s need with a single approach. Additional communications, e.g. public media campaigns, billboards, videos on social media, were also suggested as necessary. This study showed that preferences on receiving information on individual CRC risk varied substantially and no consensus was reached. Introducing a personalised screening programme will require careful communication, particularly around the rationale for the strategy, and a layered approach to deliver information.

## Highlights

• Receiving information about individual colorectal cancer risk varied widely among the target population of screening.
• It is impossible to address everyone’s need with respect to risk communication with a single approach.
• The risk information may have minimal impact on the decision to participate in personalised cancer screening.
• Implementing personalised screening requires careful communication particularly of the rationale for the strategy.
• A layered approach to deliver information on individual’s CRC risk is required**.**

## 1 Introduction

In 2014, a nationwide faecal immunochemical test (FIT)-based colorectal cancer (CRC) screening programme was initiated in the Netherlands (Toes-Zoutendijk et al., 2017). A cut-off of 47 µg Hemoglobin per gram (Hb/g) faeces is considered as positive. Positives are referred for follow-up colonoscopy and negatives are invited for repeat testing in two years. Having a faecal Hb (f-Hb) concentration just below the cut-off is associated with a higher risk for the detection of CRC and/or advanced adenomas (AA) at consecutive screening and having an interval CRC (Breekveldt et al., 2023a, Meester et al., 2023). Individuals with f-Hb concentrations close to 47 µg Hb/g faeces may therefore benefit from a shorter screening interval (i.e. increase the benefit), whereas individuals with undetectable f-Hb concentrations could benefit from a longer screening interval (i.e. decrease the harms) (Breekveldt et al., 2023b). A nationwide randomised controlled trial (RCT) is currently being carried out within the Dutch CRC screening programme to assess feasibility, acceptability and (cost-) effectiveness of such personalised screening intervals based on f-Hb concentration in those with a prior negative FIT (Breekveldt et al., 2023b).

Public preferences for cancer risk communication and acceptability of risk-stratified screening have been studied previously, mainly in the context of breast cancer screening (Henneman et al., 2011, Koitsalu et al., 2016, Meisel et al., 2015, Timmermans et al., 2008). The acceptability of risk-based screening varies. It may be acceptable by the public when the rationale behind the strategies is explained and the public can see that the strategies result in greater benefit to the population as a whole (Taylor et al., 2023). In contrast, receiving more- or less-intensive screening based on individual risk causes anxiety (Cairns et al., 2022). Explaining the benefit of risk-stratified screening in an understandable manner, especially for those receiving less-intensive screening, appears to be crucial (Piper et al., 2018). Thus, transparency and public education is required for personalised screening strategies to be acceptable to the public. Evidence on individuals’ information needs regarding risk stratification based on personal CRC risk is scarce. In this study, we aimed to gain insight into information needs to make a well-informed decision to participate in personalised CRC screening.

## 2 Methods

### 2.1 PERFECT-FIT study

The focus group was conducted as part of a nationwide mixed-method study: “Personalised CRC screening: effectiveness of tailored intervals based on prior f-Hb concentration in a FIT-based programme (PERFECT-FIT)”. The study is described in detail in the study protocol (Breekveldt et al., 2023a). In short, the aim of the PERFECT-FIT study is to evaluate the effectiveness, feasibility and acceptance of personalised CRC screening through tailored invitation intervals based on prior f-Hb concentrations; one year with f-Hb concentrations of > 15–46.9 µg Hb/g faeces, two year with f-Hb concentrations of > 0–15 µg Hb/g faeces and three years with f-Hb concentration of 0 µg Hb/g faeces. In the current uniform CRC screening programme in the Netherlands, the cut-off for a positive FIT is set at ≥ 47 µg Hb/g faeces and all individuals that tested negative are re-invited after two years, irrespective of their f-Hb concentration. At present, the target population is not informed of the quantitative amount of f-Hb concentration but only whether a follow-up colonoscopy is recommended. Anyone can request their f-Hb concentration at any time, provided they are aware of it.

The focus group in this paper, which consisted of three sessions, was conducted before the start of the national RCT. The online sessions took place between February and May 2022. The online platform Microsoft TEAMS was used. The first session was led by an experienced moderator (IK), with one expert on CRC screening (ETZ). The second and third sessions were led by ETZ, with an additional expert on CRC screening (EB). A topic guide was developed; the English translation can be found in Appendix I.

### 2.2 Study population focus group

Qualitative research methods allow for the in-depth exploration of the individual experiences and perspectives. Participants can build on the responses of each other, allowing for exploration and contradiction of individual’s perspectives. We aimed for between four to five individuals per focus group session (Dworkin, 2012, Malterud et al., 2016).

Participants were recruited through GENERO, a networking organisation for elderly people in the Southwest region in the Netherlands. Due to an insufficient number of individuals identified through GENERO for session 3, individuals were also recruited through a nationwide network for immigrants (NOOM) living in the Netherlands.

To be eligible to participate in this study, a participant had to meet all of the following inclusion criteria: eligible for CRC screening, i.e. aged 55 to 75; having provided informed consent; having access to a laptop, computer, or iPad/Tablet with camera and microphone; and Dutch language proficiency. Subjects who did not meet all the inclusion criteria were excluded from participation in this study. All participants received financial compensation for participating in the focus groups (25 euros per person).

### 2.3 Qualitative data and thematic analysis

All focus group sessions were audio recorded. The recordings were transcribed with all personal identifiers removed. The full transcripts were read by two researchers (ETZ and LdJ) to familiarise themselves with the data. Subsequently, they coded the data and generated the main themes. Only the main themes and quotations were translated from Dutch into English. Codings were discussed among the researchers and the final themes and subthemes. Coding and analyses were performed using thematic analysis approach (Braun and Clare, 2006). Data was coded and managed using NVivo software (QSR International).

### 2.4 Ethical considerations

Study participants were recruited by our contacts at GENERO and NOOM, by sending an information letter. Individuals who indicated to our contacts to be interested were contacted by one of the investigators by phone, received information about the focus groups and all of them gave their verbal consent. The study was conducted according to the principles of the Declaration of Helsinki. Ethical approval was received from the medical ethical committee of the Erasmus MC University Medical Center, Rotterdam, the Netherlands (MEC-2021-0663).

## 3 Results

A total of 14 individuals participated in the three focus groups; four to five per session. Men (50%) and women (50%) were equally represented. The median age was 69 (interquartile range 66–73 years; Table 1). Five (36%) individuals had a migrant background. Eleven participants had previously participated in the national CRC screening programme (79%). Two (14%) individuals had been diagnosed with CRC or AA through the screening programme.

**Table 1:** Demographics of study participants of the focus groups.

Gender, n (%)	
Men	7 (50)
Women	7 (50)
Age	
Median (Min-Max)	69 (66–73)
Migrant background, n (%)	
Yes	5 (36)
No	9 (69)
Participation in national CRC screening programme, n (%)	
Yes	11 (79)
No	3 (21)
CRC or AA detected through screening, n (%)	
Yes	2 (14)
No	12 (86)

Abbreviation: CRC (colorectal cancer), AA (advanced adenomas).

**Fig. 1:**
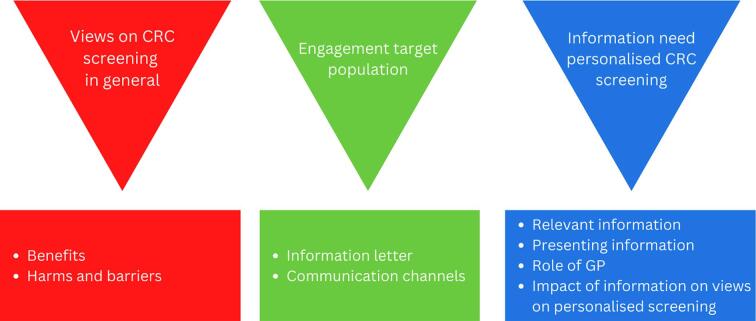
Summary of main themes and sub-themes of the focus groups on information needs on personalised risk in CRC screening. *Abbreviations: CRC, colorectal cancer; GP, general practitioner.*

Three overarching themes were identified (Fig. 1):

1) views on CRC screening in general;
2) engagement of the target population;
3) information need about personalised CRC risk and screening.

### 3.1 Views on CRC screening

#### 3.1.1 Benefits of CRC screening in general

The majority of participants understood that CRC screening leads to early detection of CRC or can even prevent CRC. A small number of participants had had a positive FIT and undergone a follow-up colonoscopy in the past. In two participants a relevant finding (CRC or AA) was detected at colonoscopy. Their experience, including the perceived benefits of CRC screening, was shared with the other participants and well-received (Table 2a).

**Table 2a:** Focus group quotations ‘Benefits of CRC screening in general’.

*“It is, of course a form of cancer that has no symptoms. So by the time you have symptoms, you are already at a fairly advanced stage. And if you can prevent that in this way [screening], yeah, it’s just a win*–*win” (Focus group 1).*

#### 3.1.2 Harms and barriers of CRC screening in general

Similar disadvantages of CRC screening or the organisation of the screening programme were addressed across all three focus groups (Table 2b). Stool collection was considered an unpleasant and complex task, although it was debated that it most likely only has a negative impact on individuals in doubt to participate. The deductible excess is an obligatory amount that first needs to be paid out of pocket before the health insurance reimburses healthcare costs. It was discussed that this might be a barrier for individuals to participate in screening, although it was felt again likely to have negative effects only on individuals in doubt to participate. Stopping screening at the age of 75 years was extensively debated; there was misunderstanding about the fact why 75-years-old individuals were no longer entitled to participate. Besides the barriers to participate in FIT-based CRC screening, harms of CRC screening were not explicitly mentioned during the focus group.

**Table 2b:** Focus group quotations ‘Harms and barriers of CRC screening in general’.

*“The collection is a hassle and I have the impression that when people are already in doubt, the whole hassle [of collecting stool], is a deciding factor not to participate” (Focus group 1).*
“*What I hear from people is that they are 76 years old and they can no longer participate. That can be explained, but it goes through people’s minds” (Focus group 1).*
*“It stops at 75, doesn’t it? The fact that you never receive an invitation again, is that because of medical reasons or is it financial?” (Focus group 2).*

### 3.2 Engagement of the target population

#### 3.2.1 Information letter

The information letter format and content were important considerations for participants; one individual had even kept his first information letter (since October 2015) (Table 2c). An important topic was language. Although the information letter refers to the website for information in different languages, the letter must first be opened and read in Dutch to find this reference to the website. It was suggested to add a small leaflet with information in several languages to make it more identifiable for migrant populations, especially because first-generation immigrants at an older age have more difficulty using the internet. Using pictures or infographics were considered helpful in understanding the information. The majority of participants that had participated in CRC screening said that they had already made the decision to participate before receiving the invitation.

**Table 2c:** Focus group quotations ‘Information letter’.

*“Yes, it is a good idea of use pictures, just like Ikea” (Focus group 2).*
“*There is some information about how to do it, but I think indeed for people, especially for our migrants who are not sufficiently proficient in the Dutch language. If you explain it with pictures, well, that will also make it clearer” (Focus group 2).*
*“I think that a purple envelope [colour of the Dutch invitation envelope] is not enough. There should be something else to make it more recognisable. Maybe more in the life-world of people, so to speak and maybe this is even more difficult for men” (Focus group 3).*
*“This is of course also an old-fashioned way of passing on this [information on CRC screening and invitation] by letter…. Maybe there are other ways as well” (Focus group 3).*

#### 3.2.2 Communication channels

A hardcopy information letter alone was considered insufficient to inform all individuals within the target population (Table 2d). They all preferred various communication channels to be informed about CRC screening. Several suggestions were made to inform and better engage the target population in CRC screening; public media campaigns, billboards, videos on social media, posters in the waiting room of the general practitioner (GP), interviews in magazines and encouragement through key figures in communities. Social media, for example Facebook, was suggested as a platform to share information through a video. This video should be available in different languages to also address the language barrier. It was pointed out that there was a public media coverage at the launch of the national CRC screening programme. This publicity was considered informative and when individuals were eventually invited, the letter came as no surprise.

**Table 2d:** Focus group quotations ‘Communications channels’.

“*Before the population screening started, there was a lot of publicity in the press. So when the invitation letter came in it complemented the whole thing. It didn’t influence my decision whether to participate or not” (Focus group 1).*
*“I have the impression that people do not read letters. The more information they [invitation letter] contain, the less people read them. So it is important that the information is also presented through advertisements or on regional or national television. The information about CRC screening is already in people’s mind and the details just need to be given in the information letter” (Focus group 3).*
*“You really need to find someone who can give you more information, so to speak. People who know how the organisation works and who know the culture and the differences*…*.*… *When you reach them, you don't have to reach out to everyone. People who really have a public function. We have to look for them” (Focus group 2).*
*“You could also use the billboards we have in the city. We have so many billboards where you can also present the information” (Focus group 2).*

Another topic that was addressed regarding communication is the use of key figures in communities to involve individuals from different cultures who may not be reached with the traditional information leaflet.

### 3.3 Information need personalised CRC screening

#### 3.3.1 Relevant information

##### 3.3.1.1 Risk communication

The PERFECT-FIT RCT on tailored invitation intervals (1, 2 or 3 years) using prior f-Hb concentration was used as an example when discussing cancer risk communication. During the sessions, it became clear that what was considered as relevant information varied substantially among focus group participants. Some participants preferred to receive detailed information on their f-Hb concentration and whether they were at higher or lower risk of developing CRC (Table 2e). Other participants clearly indicated that they preferred not to receive detailed information, but only which risk group they fall into and that they will be re-invited after a certain time interval. In all three sessions they came to the conclusion that it is probably impossible to address everyone’s needs.

**Table 2e:** Focus group quotations ‘Risk communication’.

*“I have the feeling that no matter what you write down, you will never please everyone. One person will think they are getting too much information, the other person will think they are getting too little information. One person wants the test earlier, another wants it later. We are, of course, a country of experts” (Focus group 1).*
*“I wonder if you have to give such an explanation. What I would suggest is that if you test negative two or three times, you say that the interval will be extended. That you can determine that based on your personal data. But I won’t start saying you have a little bit of blood” (Focus group 1)*
*“I actually think that if there is blood found in the stool during the population screening, but not to such an extent that it is alarming, I am shocked not to report it, I think that is actually a bit misleading. You could say in the result letter that there is indeed blood in the stool. It is not yet necessary to have a colonoscopy or something like that, but it should be monitored for this or that reasons” (Focus group 3).*

The meaning of a negative FIT was new to the participants; no communication is provided to the public on the predefined cut-off for a negative FIT. All focus group participants were unaware that having a negative FIT does not mean that there was no blood in their stool sample. Hearing that their stool may have contained blood came as a surprise to many of the study participants; one person felt misled. The response to the information that a previous negative FIT indicates that their stool may have contained blood ranged from acceptant to surprise or alarmed.

##### 3.3.1.2 Costs

During the discussion on the rationale behind shortening and lengthening the screening interval, some participants were under the impression that the decision to introduce personalised CRC was cost-driven (Table 2f). They had not appreciated that the aim of the current RCT is to improve the balance of the benefits and harms of CRC screening by intensifying screening in those at highest risk (i.e. shortening the screening interval) and lessening screening in those at lowest risk (i.e. extending the screening interval).

**Table 2f:** Focus group quotations ‘Costs’.

*“I think it’s very important, if you start with it, to do it very carefully, for example in a public campaign or I don't know what to call it. But just to clarify that [that people think it might be cost-driven] a lot” – “Yes, because it will be understood as retrenchment” (Focus group 1).*
*“What is the idea behind extending up to three years? Is it just costs, or are there other reasons?” (Focus group 3).*

#### 3.3.2 Presenting information

Similar to the discussions around the information provision on the current Dutch uniform CRC screening programme, suggestions were made to use figures or infographics to communicate different risk profiles (Table 2g). The participants also favoured layered information, with some information provided in the results letter and additional information available elsewhere for those wanting more details. This was particularly important when providing information about the amount of blood in their stool as it was felt that detailed information on this might frighten individuals. Another recommendation that recurred in all sessions was that it would be beneficial to raise public awareness before personalised screening is implemented nationally, as discussed in Section 2.2.

**Table 2g:** Focus group quotations ‘Presenting information’.

*“The best thing would be if it will be presented in different ways, so that you get repetition. Because of course people take in information in different ways” (Focus group 3).*

#### 3.3.3 Role of the general practitioner

Instead of sending information by letter, another option discussed was to refer individuals to their general practitioner (GP) (Table 2h). The GPs are aware of patients' medical records and can communicate information that is relevant to them based on their medical condition and communicate this in a way that is most likely to be understandable to individuals. Some participants said that they would contact their GP directly if they were given a 1-year interval, as they would be concerned if it indicated that they were at higher risk for CRC. Others realised that the GP could be the right person, but that GPs would have restricted time for this additional task.

**Table 2h:** Focus group quotations ‘Role of the general practitioner’.

*“Yes, but they [Population Screening Information Line] cannot, in my opinion, respond to your personal situation. The person who can do that is the doctor. So if your doctor knows the background information, he/she can give an explanation” (Focus group 1).*

#### 3.3.4 Impact of information on views on personalised screening

Shortening the invitation interval when at high risk was well-accepted and understood by the participants (Table 2i). Views on extending the invitation interval for those at lower risk for CRC were diverse. From the study participants’ perception, performing the stool test is not a harm (burden). They felt that individuals who have already decided to participate accept harms involved in screening and to them there is no benefit in extending the interval to three years. To them, it is better to choose the safer option than the riskier one. However, not all participants were negative about extending the interval, as some believed they were in good health and did not need more intensive screening.

**Table 2i:** Focus group quotations ‘Impact of information on views on personalised screening’.

*“But I think it is better to have one too many than one too few” (Focus group 3).*
*“Yes, I agree, because I think that you should stick to the two years*…*.*…*. If you have to wait three years for the next screen, people think it will be much too late. I don't know how aggressive this cancer is, I have no idea” (Focus group 3).*
*“No, I would not mind [3-year interval]. If I am so healthy that they do not want to see me three years I will explain that as something positive” (Focus group 2).*
“*I think that at some point people will be willing to participate in screening, that they will take the risk of that tension. And then it makes absolutely no difference whether it is every three years or every two years” (Focus group 3).*

Focus group participants clearly stated that they would participate in personalised screening, regardless of whether the information presented met their needs. This was due to their positive view on CRC screening in general and belief that CRC screening will lead to benefits. The participants that had not participated in CRC screening before, said they would reconsider their choice to not participate, as a result of the discussion during the focus group.

## 4 Discussion

In this study we gained insights into information needs regarding risk communication in personalised CRC screening. No consensus was reached during the focus group on the preferred method for communicating individuals’ CRC risk. Several suggestions were made, which ranged from “I want to know everything” to “I only want to know which risk group I am in”.

The variation is in line with findings of other studies which have shown that the presentation of risk in a single format is not optimal (Dorval et al., 2013, Hill et al., 2010). In a study on optimal communication about breast cancer risk, women’s preferences varied from preferring not to be given detailed information to the more detailed information on individual breast cancer risk (Dorval et al., 2013). In another study on risk communication of cardiovascular disease, it was also concluded that a combination of different formats of risk communication is preferred (Hill et al., 2010). Our findings reaffirm that it will be challenging to address everyone’s needs and a layered approach to deliver information on individual’s CRC risk is required. Different formats need to be designed and evaluated in larger cohorts.

The findings of this qualitative study emphasise that the public particularly need understandable information on the balance between the harms and benefits of CRC screening, given that personalised screening aims to improve this balance. Increasing the benefits by intensifying screening was well-accepted among our participants, but lessening screening to reduce the harms of screening was received differently. This is consistent with the findings of previous research, in which it has been shown that lessening screening was not accepted by the public and highlights further the importance of clearly communicating the rationale and evidence behind the personalised approach (Ghanouni et al., 2020, Lippey et al., 2019, Meisel et al., 2015, Piper et al., 2018, Rainey et al., 2020). Explaining these benefits is also essential to avoid that the general perception will be that optimising CRC screening is only cost-driven. The discussion on stopping age of screening was beyond our research scope, but gave insight in the issue of informing the population about the optimal balance between harms and benefits of screening. The stopping age was chosen based on the harm/benefit ratio of CRC screening per age (Health council of the Netherlands, 2013). This optimal harm/benefit ratio may however be perceived differently by the target population, having another view on the benefit and especially the harms of screening at an older age (Degeling et al., 2018, Piper et al., 2018). The public seems not well informed and may disagree with the rationale for stopping CRC screening at the age of 75, similar to the disagreement with the rationale for lengthening the screening interval to reduce potential harms of screening.

Individuals that previously participated in CRC screening indicated that they had already made the decision to participate before receiving their invitation letter. Moreover, the indicated that they would participate in personalised CRC screening, regardless of whether CRC risk communication met their preference. This is in line with previous research, in which participants reported that receiving a low risk estimate would have no impact on whether they chose to participate, while receiving a high risk might have a positive impact (Usher-Smith et al., 2021a). Literature consistently showed that the concept of personalised screening seems to have no negative impact on individuals’ view on cancer screening (Scherer et al., 2019, Usher-Smith et al., 2021a, Usher-Smith et al., 2021b). We can carefully conclude that individuals also seem to accept new screening strategies if they are positive towards uniform CRC screening. Further research is needed to examine whether engaging individuals in CRC screening in general might actually be more important than addressing everyone’s need in communication of personal’ CRC risk.

Focus group participants shared their views on the minimum requirements for informing and engaging the target population in a personalised CRC screening programme. The organisational structure may already be optimal: sending a pre-invitation letter, then mailing an invitation including a test kit and a reminder letter if necessary (Tinmouth et al., 2022, Toes-Zoutendijk et al., 2020). Despite the success of the media campaign when CRC screening started in 2014, focus group participants indicated that there is no general awareness of the CRC screening programme at present and a hardcopy letter is insufficient. Especially relevant, as it is known that non-participants read no information (Kobayashi et al., 2016, Usher-Smith et al., 2021b). A media campaign accompanying the introduction of a personalised screening programme could therefore potentially raise the public awareness of the personalised approach before participation (Thomas et al., 2022). Other suggestions to raise awareness were information leaflets in different languages, infographics, social media, national campaigns, billboards, interviews in magazines, and key figures in the community.

The main strength of our study was using focus groups rather than interviews which gave the benefit of providing a way for participants to build on each other's responses and consider aspects that they might not have considered themselves. This was particularly important around the variation in preferences for information, only by the group discussion we became aware that there is not one preferred format. Another strength was the inclusion of individuals who had previously chosen not to take part in screening and thereby we were able to capture the views of a hard-to-reach group. In line with this, the participants were diverse in terms of gender and migrant background, the result of recruiting the participants through the elderly network within a large multicultural city. Lastly, the personalised CRC screenings strategy discussed in the focus group was not a hypothetical scenario, but based on real scenario of a nationwide RCT (Breekveldt et al., 2023b). Our method of recruitment - through an elderly network – is also a limitation and may have introduced some selection bias; participants were relatively old (69 years) and did not cover the full age range (55–75) of the screening programme. The lack of younger individuals in this study sample may have influenced the results of the study. Younger people may have had different information preferences, using different types of social media or communicating their individual CRC risk. However, this is in line with our conclusion; that communication should happen using a layered approach and through multiple channels. Also few individuals had been diagnosed with CRC or a precancerous stage, and these patients may have a more positive view towards CRC screening in general. Another limitation was that not all participants were ready for the discussion on personalised screening because they had outstanding questions on the CRC screening programme in general. Positively, this enabled us to obtain relevant insights that can be useful for communication methods within the current uniform CRC screening programme.

In conclusion, this study showed that preferences for receiving information about individual CRC risk varied widely and no consensus was reached. A layered approach to deliver information is required. Nevertheless, the provision of information may have minimal impact on the decision to participate in personalised CRC screening.

## Authors role

ETZ and ILV acquired funding for the project. ETZ, IK, EB were responsible for data collection. ETZ and LdJ carried out the formal analysis. ETZ and BD were responsible for the methodology. ETZ, LdJ and BD drafted the original version of the manuscript. All authors revised and edited the original and final versions of the manuscript. ILV was responsible for project supervision.

## Funding

The project was funded by Maag-Lever-Darm Stichting, project number WO-1944. JUS and BD are funded by an NIHR Advanced Fellowship award (NIHR300861).

## Ethical approval

Ethical approval was received from the Erasmus MC University Medical Centre (MEC-2021-0663).

## Declaration of Competing Interest

The authors declare that they have no known competing financial interests or personal relationships that could have appeared to influence the work reported in this paper.

## Data Availability

The data that has been used is confidential.

## APPENDIX I: Questionnaire for the semi-structured focus group (translation of original Dutch questionnaire)

### Theme I: Bowel cancer population screening

1. What do you already know about the Dutch uniform colorectal cancer (CRC) screening programme?
  a. Have you participated in CRC screening before?
  b. What are reasons to participate in CRC screening?
2. Can you take me along in your choice process to participate in CRC screening?
  a. Did you discuss your choice to participate with anyone else?
  b. Did you inform or visit your GP?
  c. Did you read the invitation letter?
- If yes:
What did you found difficult when reading the information about the CRC screening programme?
What did you found helpful when reading the information about the CRC screening programme?

- If not, why not?  *In the current CRC screening programme you will receive a stool test at home every two years. You can perform this at home and send it back by post. The laboratory then checks whether there was blood in your stool, which is often invisible. With the stool test you can have a favourable or unfavourable result. If the result is unfavourable, the lab (laboratory) found blood in your stool. Further research is then required. With a favourable result, the laboratory found little or no blood in your stool. This means that no further investigation is required. Until now, all people with a favourable result were invited again every two years. However, the risk of CRC differs in people who previously had a favourable result. People without blood in the stool have a lower risk of CRC than people with very little or little blood, even though this risk is still very low. In CRC screening based on individual risk, people with little blood are invited earlier than people without blood. This means that not everyone is invited every two years with a favourable result, but the screening interval is based on personal risk.*

3. Do you think that information about the results and personal risk can influence people's opinions about the CRC screening programme, and if so, how?
  a. Now you have the knowledge about the cut-off value and a favourable result, do you prefer to know that a favourable result does not always mean that no blood was found in the stool?
  b. What is your response or feeling that, despite a favourable result, there may have been a little blood in your stool?
  c. Would you have needed more information about a favourable result?
  d. If so, what would you like to have known before participating?

### Theme II: Personalized colorectal cancer screening

*We are conducting a scientific study with people who had a favourable result in the previous screening round. However, there is a difference in CRC risk among individuals with a prior favourable result. In the study, we do not invite all individuals after 2 years as standard, but after one, two or three years. Depending on whether people had no blood (3 years), very little blood (2 years) or little blood (1 year) in the stool. The majority of people have no blood in their stool (85%). In the context of this study, we would like to find out what information people consider desirable in order to make a well-informed choice about participation in a population CRC screening based on personal risk.*

4. The above information is new to you, as it was previously communicated that there was no blood in your stool. Do you have questions about this?
5. Would you participate in CRC screening on personal risk?
  a. What are reasons for not participating?
  b. What is a reason to participate
6. What information do you think is necessary to make a good choice about participation in a CRC screening based on personal risk?
  a. How would you best understand information from, for example, the invitation folder regarding a higher or lower risk of colorectal cancer?
  b. What information is helpful in making a good choice regarding participation in CRC screening using personal risk?
  c. How would you like to get this information presented? (Short folder, letter, website, film)?
7. How do you feel about some people being invited after 1 year, and the majority of people after 3 years
8. Does anyone have anything to add that hasn't been discussed?
